# Supplementary material for: First Diagnostic Questionnaire for Assessing Patients’ Social Functioning: Comprehensive DDX3X Syndrome Patient Profile
Source: J Clin Med. 2024 Dec 22;13(24):7842. doi: 10.3390/jcm13247842 (PMC11676840; doi:10.3390/jcm13247842)
Supplement: Supplementary file 1 [file jcm-13-07842-s001.zip › 07.08.2024 Supplementary 1 - Supplementary tables.pdf]

Table S1. Comparison of social skills domain (SS) between children with autism spectrum disorders and DDX3X syndrome.

		ASD	DDX3X	φ_c	OR	95% CI	X^2	*p*-value	*p*-value corr#	
SS1	No	10	23	-.096081	0.64	0.27-1.52	1.033934	.03924	.08502	1
	Yes	32	47							
SS2	No	13	7	.2648489	4.04	1.46-11.17	7.621336	.00577	.01875	2
	Yes	29	63							
SS3	No	5	2	.1809524	4.59	0.85-24.85	2.285714	.06759	.12552	4t
	Yes	37	68							
SS4	No	12	5	.2891217	5.20	1.68-16.09	9.098629	.00256	.01109	2
	Yes	30	65							
SS5	No	35	26	.4490308	8.46	3.29-21.78	22.58241	<.00001	.00013	1
	Yes	7	44							
SS6	No	32	36	.2454578	3.02	1.29-7.08	6.747950	.00939	.02441	1
	Yes	10	34							
SS8	No	10	0	.4042260	N/A	N/A	15.48967	.00003	.00002	3
	Yes	32	70							
SS9	No	26	51	.1143939	1.65	0.73-3.73	1.465628	.22604	.36732	1
	Yes	16	19							
SS10	No	25	46	-.062212	0.77	0.35-1.69	0.433482	.51029	.60307	1
	Yes	17	24							
SS11	No	5	9	-.013941	0.92	0.29-2.94	0.021893	.88237	.88237	2
	Yes	37	61							
SS12	No	22	29	.1064712	1.56	0.72-3.36	1.269645	.25983	.37531	1
	Yes	20	41							
SS13	No	16	30	.0468601	1.22	0.56-2.66	0.245938	.61995	.67161	1
	Yes	26	40							
SS14	No	10	22	.0816497	1.47	0.61-3.50	0.746667	.38753	.50379	1
	Yes	32	48							

Legend: ASD – a group of children with autism spectrum disorders (DDX3X syndrome negative), DDX3X & DDX3X+ASD – DDX3X syndrome affected group (children with DDX3X syndrome without and with ASD), # Benjamini-Hochberg correction for multiple comparisons, [1] Pearson's χ^2, [2] V-square correction, [3] Yates correction, [4] Fisher's exact test, N/A – not available, φ_c – Cramér's phi, OR – odds ratio, CI – confidence interval, [t] tendency ($p = 0.05 – 0.1$); *p*-values < 0.05 are indicated in bold.

Table S2. Comparison of play domain (PD) between children with autism spectrum disorders and DDX3X syndrome.

		ASD	DDX3X	φ_c	OR	95% CI	χ^2	*p*-value	*p*-value corr[#]	
PD1	No	24	35	.0692599	1.33	0.62-2.88	0.537256	.46357	.49923	1
	Yes	18	35							
PD2	No	10	6	.2108185	3.33	1.11-9.99	4.809422	.02830	.04402	2
	Yes	32	64							
PD3	No	9	4	.2375084	4.50	1.29-15.70	4.879150	.01473	.04124	3
	Yes	33	66							
PD4	No	9	6	.1827623	2.91	0.95-8.87	3.612419	.05735	.08029	2t
	Yes	33	64							
PD5	No	15	12	.2101975	2.68	1.11-6.51	4.948497	.02611	.04402	1
	Yes	27	58							
PD6	No	11	5	.2635231	4.61	1.47-14.43	7.538100	.00604	.02819	2
	Yes	31	65							
PD7	No	16	13	.2157745	2.70	1.13-6.42	5.214569	.02240	.04402	1
	Yes	26	57							
PD8	No	15	36	.1527630	1.91	0.87-4.18	2.613693	.10594	.13483	1
	Yes	27	34							
PD9	No	28	21	.3578300	4.67	2.06-10.60	14.34074	.00015	.00210	1
	Yes	14	49							
PD10	No	17	13	.2394664	2.98	1.26-7.06	6.422547	.01127	.03945	1
	Yes	25	57							
PD11	No	20	27	.0887569	1.45	0.67-3.14	0.882313	.34757	.40550	1
	Yes	22	43							
PD12	No	20	31	.0324043	1.14	0.53-2.46	0.117604	.73165	.73165	1
	Yes	22	39							
PD13	No	20	18	.2239776	2.63	1.17-5.90	5.618587	.01777	.04146	1
	Yes	22	52							
PD14	No	5	25	.2602896	4.11	1.43-11.80	7.588076	.00588	.02819	1
	Yes	37	45							

Legend: ASD – a group of children with autism spectrum disorders (DDX3X syndrome negative), DDX3X & DDX3X+ASD – DDX3X syndrome affected group (children with DDX3X syndrome without and with ASD), [#] Benjamini-Hochberg correction for multiple comparisons, [1] Pearson's χ^2, [2] V-square correction, [3] Yates correction, N/A – not available, φ_c – Cramér's phi, OR – odds ratio, CI – confidence interval, [t] tendency (p = 0.05 – 0.1); *p*-values < 0.05 are indicated in bold.

Table S3. Comparison of communication domain (CD) between children with autism spectrum disorders and DDX3X syndrome.

		ASD	DDX3X	φ_c	OR	95% CI	χ^2	*p*-value	*p*-value corr[#]	
CD1	No	20	34	.0092273	1.04	0.48-2.23	.0095360	.92221	.92221	[1]
	Yes	22	36							
CD2	No	1	1	.0289122	1.38	0.08-23.10	N/A	.82425	.80654	[4]
	Yes	24	33							
CD3	No	6	6	.0894427	1.78	0.53-5.92	.3982222	.36086	.72172	[3]
	Yes	36	64							
CD4	No	4	12	.1054093	1.97	0.59-6.55	1.308525	.25266	.72172	[2]
	Yes	38	58							
CD5	No	27	29	.2213133	2.54	1.15-5.61	5.485714	**.01917**	.11502	[1]
	Yes	15	41							
CD6	No	13	18	.0566792	1.29	0.55-3.02	.3598035	.54862	.82293	[1]
	Yes	29	52							

Legend: ASD – a group of children with autism spectrum disorders (DDX3X syndrome negative), DDX3X & DDX3X+ASD – DDX3X syndrome affected group (children with DDX3X syndrome without and with ASD), [#] Benjamini-Hochberg correction for multiple comparisons, [1] Pearson's χ^2, [2] V-square correction, [3] Yates correction, [4] Fisher's exact test, N/A – not available, φ_c – Cramér's phi, OR – odds ratio, CI – confidence interval, [t] tendency (p = 0.05 – 0.1); *p*-values < 0.05 are indicated in bold.

Table S4. Comparison of social skills domain (SS) between children with autism spectrum disorders and DDX3X syndrome with facial dysmorphia.

		ASD	DDX3X	φ_c	OR	95% CI	X^2	*p*-value	*p*-value corr[#]	
SS1	No	2	7	.2359254	4.29	0.33-55.58	N/A	.28696	.62493	[4]
	Yes	1	15							
SS2	No	0	3	-.161165	N/A	N/A	N/A	.28962	.62493	[4]
	Yes	4	18							
SS3	No	2	0	.7985494	N/A	N/A	N/A	**.00147**	**.01764**	[4]
	Yes	1	22							
SS4	No	0	3	-.136364	N/A	N/A	N/A	.36496	.62493	[4]
	Yes	3	19							
SS5	No	3	8	.4165978	N/A	N/A	N/A	.07174	.43044	[4t]
	Yes	0	14							
SS6	No	2	13	.0502519	1.38	0.11-17.67	N/A	.65435	.71304	[4]
	Yes	1	9							
SS8	No	0	0	N/A	N/A	N/A	N/A	N/A	-	-
	Yes	3	22							
SS9	No	1	14	.2010076	3.50	0.27-44.95	N/A	.34565	.62493	[4]
	Yes	2	8							
SS10	No	3	13	.2769558	N/A	N/A	N/A	.24348	.62493	[4]
	Yes	0	9							
SS11	No	1	3	.1745950	3.12	0.21-46.73	N/A	.42174	.62493	[4]
	Yes	2	19							
SS12	No	2	10	.1379729	2.40	0.189-30.52	N/A	.46870	.62493	[4]
	Yes	1	12							
SS13	No	1	8	.0205152	1.14	0.09-14.68	N/A	.71304	.71304	[4]
	Yes	2	14							
SS14	No	1	7	-.010555	0.93	0.07-12.10	N/A	.70435	.71304	[4]
	Yes	2	17							

Legend: ASD – a group of children with autism spectrum disorders (DDX3X syndrome negative), DDX3X & DDX3X+ASD – DDX3X syndrome affected group (children with DDX3X syndrome without and with ASD), [#] Benjamini-Hochberg correction for multiple comparisons, [4] Fisher's exact test, N/A – not available, φ_c – Cramér's phi, OR – odds ratio, CI – confidence interval, [t] tendency (*p* = 0.05 – 0.1); *p*-values < 0.05 are indicated in bold.

Table S5. Comparison of play domain (PD) between children with autism spectrum disorders and DDX3X syndrome with facial dysmorphia.

		ASD	DDX3X	φ_c	OR	95% CI	X^2	*p*-value	*p*-value corr[#]	
PD1	No	3	12	.3015113	N/A	N/A	N/A	.19783	.36812	[4]
	Yes	0	10							
PD2	No	1	2	.2424242	5.00	0.30-82.74	N/A	.28924	.36812	[4]
	Yes	2	20							
PD3	No	1	1	.3448282	10.5	0.49-239.78	N/A	.15903	.36812	[4]
	Yes	2	21							
PD4	No	1	0	.5527708	N/A	N/A	7.638889	**.00571**	.07994	[4]
	Yes	2	22							
PD5	No	2	4	.3689144	9.00	0.65-125.32	N/A	.13261	.36812	[4]
	Yes	1	18							
PD6	No	1	2	.2424242	5.00	0.30-82.74	N/A	.28924	.36812	[4]
	Yes	2	20							
PD7	No	2	6	.2744302	5.33	0.40-70.19	N/A	.23130	.36812	[4]
	Yes	1	16							
PD8	No	1	12	.1379729	2.40	0.19-30.52	N/A	.46870	.50435	[4]
	Yes	2	10							
PD9	No	3	8	.4165978	N/A	N/A	N/A	.07174	.36812	[4t]
	Yes	8	14							
PD10	No	1	4	.1230915	2.25	0.16-31.33	N/A	.50435	.50435	[4]
	Yes	2	18							
PD11	No	2	7	.2359254	4.29	0.33-55-58	N/A	.28696	.36812	[4]
	Yes	1	15							
PD12	No	2	10	.1379729	2.40	0.19-30.52	N/A	.46870	.50435	[4]
	Yes	1	12							
PD13	No	2	7	.2359254	4.28	0.33-55.59	N/A	.28696	.36812	[4]
	Yes	1	15							
PD14	No	0	10	.3015113	N/A	N/A	N/A	.19783	.36812	[4]
	Yes	3	12							

Legend: ASD – a group of children with autism spectrum disorders (DDX3X syndrome negative), DDX3X & DDX3X+ASD – DDX3X syndrome affected group (children with DDX3X syndrome without and with ASD), [#] Benjamini-Hochberg correction for multiple comparisons, [4] Fisher's exact test, N/A – not available, φ_c – Cramér's phi, OR – odds ratio, CI – confidence interval, [t] tendency (p = 0.05 – 0.1); *p*-values < 0.05 are indicated in bold.

Table S6. Comparison of communication domain (CD) between children with autism spectrum disorders and DDX3X syndrome with facial dysmorphia.

		ASD	DDX3X	φ_c	OR	95% CI	X^2	*p*-value	*p*-value corr[#]	
CD1	No	0	13	.3843531	N/A	N/A	N/A	.09565	.15942	[4t]
	Yes	3	9							
CD2	No	0	0	N/A	N/A	N/A	N/A	N/A	N/A	[4]
	Yes	0	13							
CD3	No	2	2	.5103545	20.00	1.21-330.95	N/A	.02912	.13043	[4]
	Yes	1	20							
CD4	No	0	4	.1611646	N/A	N/A	N/A	.42034	.42034	[4]
	Yes	3	18							
CD5	No	2	9	.1686229	2.89	0.22-36.87	N/A	.40652	.42034	[4]
	Yes	1	13							
CD6	No	3	0	.4522670	N/A	N/A	N/A	.05217	.13043	[4t]
	Yes	7	15							

Legend: ASD – a group of children with autism spectrum disorders (DDX3X syndrome negative), DDX3X & DDX3X+ASD – DDX3X syndrome affected group (children with DDX3X syndrome without and with ASD), [#] Benjamini-Hochberg correction for multiple comparisons, [4] Fisher's exact test, N/A – not available, OR – odds ratio, CI – confidence interval, [t] tendency (p=0.05 – 0.1); *p*-values < 0.05 are indicated in bold.

Table S7. Comparison of social skills domain (SS) between children with autism spectrum disorders and DDX3X syndrome with microcephaly.

		ASD	DDX3X	φ_c	OR	95% CI	X^2	*p*-value	*p*-value corr#	
SS1	No	0	6	-.184637	N/A	N/A	N/A	.64706	.94118	4
	Yes	1	10							
SS2	No	1	1	.6846532	N/A	N/A	N/A	.11765	.70590	4
	Yes	0	15							
SS3	No	0	1	-.062500	N/A	N/A	N/A	.94118	.94118	4
	Yes	1	15							
SS4	No	1	0	.899999	N/A	N/A	N/A	.05327	.63924	4t
	Yes	0	16							
SS5	No	1	8	.2357023	N/A	N/A	N/A	.52941	.94118	4
	Yes	0	8							
SS6	No	1	8	.2357023	N/A	N/A	N/A	.52941	.94118	4
	Yes	0	8							
SS8	No	0	0	N/A	N/A	N/A	N/A	N/A	-	-
	Yes	1	16							
SS9	No	1	11	-.161374	N/A	N/A	N/A	.70588	.94118	4
	Yes	0	5							
SS10	No	1	12	.1386750	N/A	N/A	N/A	.76471	.94118	4
	Yes	0	4							
SS11	No	0	2	-.091287	N/A	N/A	N/A	.88235	.94118	4
	Yes	1	14							
SS12	No	1	7	.2651650	N/A	N/A	N/A	.47059	.94118	4
	Yes	0	9							
SS13	No	0	4	.1386750	N/A	N/A	N/A	.76471	.94118	4
	Yes	1	12							
SS14	No	0	3	.1157275	N/A	N/A	N/A	.82353	.94118	4
	Yes	13	3							

Legend: ASD – a group of children with autism spectrum disorders (DDX3X syndrome negative), DDX3X & DDX3X+ASD – DDX3X syndrome affected group (children with DDX3X syndrome without and with ASD), # Benjamini-Hochberg correction for multiple comparisons, 4 Fisher's exact test, N/A – not available, φ_c – Cramér's phi, OR – odds ratio, CI – confidence interval, t tendency (p = 0.05 – 0.1)

Table S8. Comparison of play domain (SS) between children with autism spectrum disorders and DDX3X syndrome with microcephaly.

		ASD	DDX3X	φ_c	OR	95% CI	X^2	*p*-value	*p*-value corr[#]	
PD1	No	1	9	.2091650	N/A	N/A	N/A	.58824	.93464	4
	Yes	0	7							
PD2	No	0	1	-.062500	N/A	N/A	N/A	.94118	.94118	4
	Yes	1	15							
PD3	No	0	1	-.062500	N/A	N/A	N/A	.94118	.94118	4
	Yes	1	15							
PD4	No	0	0	N/A	N/A	N/A	N/A	N/A	-	-
	Yes	1	16							
PD5	No	0	2	-.091287	N/A	N/A	N/A	.88235	.94118	4
	Yes	1	14							
PD6	No	1	1	.6846532	N/A	N/A	N/A	.11765	.93464	4
	Yes	0	15							
PD7	No	0	4	-.138675	N/A	N/A	N/A	.76471	.94118	4
	Yes	1	12							
PD8	No	0	10	.2988071	N/A	N/A	N/A	.41176	.93464	4
	Yes	1	6							
PD9	No	1	6	.2988071	N/A	N/A	N/A	.41176	.93464	4
	Yes	0	10							
PD10	No	1	2	.5400617	N/A	N/A	N/A	.17647	.93464	4
	Yes	0	14							
PD11	No	0	9	-.265165	N/A	N/A	N/A	.47059	.93464	4
	Yes	1	7							
PD12	No	1	10	.1846372	N/A	N/A	N/A	.64706	.93464	4
	Yes	0	6							
PD13	No	1	5	.3385016	N/A	N/A	N/A	.35294	.93464	4
	Yes	0	11							
PD14	No	0	8	.2357023	N/A	N/A	N/A	.52941	.93464	4
	Yes	1	8							

Legend: ASD – a group of children with autism spectrum disorders (DDX3X syndrome negative), DDX3X & DDX3X+ASD – DDX3X syndrome affected group (children with DDX3X syndrome without and with ASD), [#] Benjamini-Hochberg correction for multiple comparisons, [4] Fisher's exact test, N/A – not available, φ_c – Cramér's phi, OR – odds ratio, CI – confidence interval, [t] tendency (p = 0.05 – 0.1)

Table S9. Comparison of communication domain (SS) between children with autism spectrum disorders and DDX3X syndrome with microcephaly.

		ASD	DDX3X	φ_c	OR	95% CI	X^2	*p*-value	*p*-value corr#	
CD1	No	1	9	-.209165	N/A	N/A	N/A	.58824	.84789	4
	Yes	0	7							
CD2	No	0	1	-.111111	N/A	N/A	N/A	.84789	.84789	4
	Yes	1	8							
CD3	No	0	0	N/A	N/A	N/A	N/A	N/A	N/A	4
	Yes	1	16							
CD4	No	0	4	.1386750	N/A	N/A	N/A	.76471	.84789	4
	Yes	1	12							
CD5	No	1	11	.1613743	N/A	N/A	N/A	.70588	.84789	4
	Yes	0	5							
CD6	No	0	7	-.209165	N/A	N/A	N/A	.58824	.84789	4
	Yes	1	9							

Legend: ASD – a group of children with autism spectrum disorders (DDX3X syndrome negative), DDX3X & DDX3X+ASD – DDX3X syndrome affected group (children with DDX3X syndrome without and with ASD), # Benjamini-Hochberg correction for multiple comparisons, 4 Fisher's exact test, N/A – not available, φ_c – Cramér's phi, OR – odds ratio, CI – confidence interval

Table S10. Comparison of social skills domain (SS) between analyzed children in different languages.

		PL	FR	EN	φ_c	X^2	*p*-value	*p*-value corr[#]	*p*-value sub-differences[&]	
SS1	No	10	10	13	.1449822	2.354223	.30817	.50078	-	1
	Yes	25	14	40						
SS2	No	11	4	5	.2496703	6.981548	.03048	.07469	PL vs EN .0088	1
	Yes	24	20	48						
SS3	No	4	3	0	.2452265	6.735238	.03447	.07469	PL vs EN .0459<br>FR vs EN .0466	2
	Yes	31	21	53						
SS4	No	11	4	2	.3350703	12.57448	.00186	.01209	PL vs EN .0003	1
	Yes	24	20	51						
SS5	No	26	8	27	.3007517	10.13058	.00631	.02734	PL vs EN .0285<br>PL vs FR .0018	1
	Yes	9	16	26						
SS6	No	28	12	28	.2671615	7.994027	.01837	.05970	PL vs EN .0095<br>PL vs FR .0154	1
	Yes	7	12	25						
SS8	No	10	0	0	.4644204	24.15686	.00001	.00013	PL vs EN .0001<br>PL vs FR .0117	2
	Yes	25	24	53						
SS9	No	24	20	33	.1745917	3.414013	.18141	.33690	-	1
	Yes	11	4	20						
SS10	No	20	17	34	.1024177	1.174811	.55577	.67042	-	1
	Yes	15	7	19						
SS11	No	3	4	7	.0895967	0.899089	.65639	.71109	-	1
	Yes	32	20	46						
SS12	No	16	12	23	.0509845	0.291135	.86453	.86453	-	1
	Yes	19	12	30						
SS13	No	16	11	19	.1006150	1.133819	.56728	.67042	-	1
	Yes	19	13	34						
SS14	No	9	5	18	.1194812	1.598884	.44958	.64939	-	1
	Yes	26	19	35						

Legend: PL – Polish, FR – French, EN – English, [#] Benjamini-Hochberg correction for multiple comparisons, [&] uncorrected, [1] Pearson's χ^2, [2] Fisher's χ^2, φ_c – Cramér's phi, OR – odds ratio, CI – confidence interval; *p*-values < 0.05 are indicated in bold.

Table S11. Comparison of play domain (PD) between analyzed children in different languages.

		PL	FR	EN	φ_c	X^2	*p*-value	*p*-value corr[#]	*p*-value sub-differences[&]	
PD1	No	19	22	18	.4443966	22.11869	.00002	.00028	PL vs FR .0022<br>FR vs EN <.0001	1
	Yes	16	2	35						
PD2	No	5	4	7	.0379645	0.161426	.92245	.92245	-	1
	Yes	30	20	46						
PD3	No	5	5	3	.1904592	4.062766	.14226	.28452	-	2
	Yes	30	19	50						
PD4	No	7	2	6	.1350630	2.043106	.41905	.53334	-	2
	Yes	28	22	47						
PD5	No	11	3	13	.1580547	2.797905	.24685	.38399	-	1
	Yes	24	21	40						
PD6	No	9	4	3	.2511504	7.06457	.02924	.10234	PL vs EN .0107	1
	Yes	26	20	50						
PD7	No	11	6	12	.0876738	0.86091	.65021	.70023	-	1
	Yes	24	18	41						
PD8	No	16	14	21	.1443138	2.332566	.31152	.43613	-	1
	Yes	19	10	32						
PD9	No	21	9	19	.2212166	5.480922	.06454	.18071	-	1t
	Yes	14	15	34						
PD10	No	14	6	10	.2080892	4.849725	.08849	.20648	-	1t
	Yes	21	18	43						
PD11	No	15	12	20	.0962182	1.03689	.59545	.69469	-	1
	Yes	20	12	33						
PD12	No	12	11	28	.1615791	2.924074	.23176	.38399	-	1
	Yes	23	13	25						
PD13	No	15	13	10	.3132831	10.99239	.00410	.01913	PL vs EN .0146<br>FR vs EN .0017	1
	Yes	20	11	43						
PD14	No	3	5	22	.3302248	12.21342	.00223	.01561	PL vs EN .0008	1
	Yes	32	19	31						

Legend: PL – Polish, FR – French, EN – English, [#] Benjamini-Hochberg correction for multiple comparisons, & uncorrected, [1] Pearson's χ^2, [2] Fisher's χ^2, φ_c – Cramér's phi, OR – odds ratio, CI – confidence interval, [t] tendency (p = 0.05 – 0.1); *p*-values < 0.05 are indicated in bold.

Table S12. Comparison of communication domain (CD) between analyzed children in different languages.

		PL	FR	EN	φ_c	X^2	*p*-value	*p*-value corr#	*p*-value sub-differences&	
CD1	No	22	10	22	.1975740	4.371974	.11237	.28452	-	1
	Yes	13	14	31						
CD2	No	1	1	0	.1912386	4.062766	.14226	.28452	-	2
	Yes	25	9	23						
CD3	No	2	2	8	.1375741	2.119782	.41392	.44816	-	2
	Yes	33	22	45						
CD4	No	2	4	10	.1669023	3.119916	.21014	.31521	-	1
	Yes	33	20	43						
CD5	No	23	11	22	.2144784	5.152111	.07607	.28452	-	1t
	Yes	12	13	31						
CD6	No	8	9	14	.1197180	1.60523	.44816	.44816	-	1
	Yes	27	15	39						

Legend: PL – Polish, FR – French, EN – English, # Benjamini-Hochberg correction for multiple comparisons, & uncorrected,
[1] Pearson's χ^2, [2] Fisher's χ^2, φ_c – Cramér's phi, OR – odds ratio, CI – confidence interval, [t] tendency (*p*=0.05 – 0.1)

Table S13. Comparison of social skills domain (SS) between analyzed children in different languages in group with ASD.

		PL	FR	EN	φ_c	χ^2	*p*-value	*p*-value corr[#]	*p*-value sub-differences[&]	
SS1	No	8	1	1	.1844727	1.429267	.48937	.80076	-	2
	Yes	21	2	9						
SS2	No	10	1	2	.1325888	0.738352	.85872	.99990	-	2
	Yes	19	2	8						
SS3	No	3	2	0	.4878773	9.997018	**.00674**	.08772	FR vs EN **.0038**	2
	Yes	26	1	10						
SS4	No	11	1	0	.3545004	5.278161	.06627	.21538	-	2t
	Yes	18	2	10						
SS5	No	25	3	7	.2210588	2.052414	.37493	.80076	-	2
	Yes	4	0	3						
SS6	No	25	1	6	.3806754	6.086379	**.04768**	.21538	PL vs FR .0826	2
	Yes	4	2	4						
SS8	No	10	0	0	.3742809	5.883621	.05277	.21538	-	2t
	Yes	19	3	10						
SS9	No	18	1	7	.1770578	1.316678	.59017	.85247	-	2
	Yes	11	2	3						
SS10	No	18	2	5	.1110525	0.517972	.87162	.99990	-	2
	Yes	11	1	5						
SS11	No	3	1	1	.1835753	1.415396	.49278	.80076	-	2
	Yes	26	2	9						
SS12	No	15	2	5	.0806517	0.273197	>.9999	>.99990	-	2
	Yes	14	1	5						
SS13	No	11	1	4	.0325731	0.044562	>.9999	>.99990	-	2
	Yes	18	2	6						
SS14	No	8	1	1	.1844727	1.429267	.48937	0.80076	-	2
	Yes	21	2	9						

Legend: PL – Polish, FR – French, EN – English, [#] Benjamini-Hochberg correction for multiple comparisons, & uncorrected, [1] Pearson's χ^2, [2] Fisher's χ^2, φ_c – Cramér's phi, OR – odds ratio, CI – confidence interval, [t] tendency (p = 0.05 – 0.1); *p*-values < 0.05 are indicated in bold.

Table S14. Comparison of play domain (PD) between analyzed children in different languages in group with ASD.

		PL	FR	EN	φ_c	χ^2	*p*-value	*p*-value corr[#]	*p*-value sub-differences[&]	
PD1	No	18	3	3	.3633734	5.545690	.06248	.29157	-	[2t]
	Yes	11	0	7						
PD2	No	4	2	4	.3806754	6.086379	**.04768**	.29157	PL vs FR .0826	[2]
	Yes	25	1	6						
PD3	No	5	2	2	.3070819	3.960571	.13803	.38122	-	[2]
	Yes	24	1	8						
PD4	No	5	1	3	.1535977	0.990874	.62309	.82806	-	[2]
	Yes	24	2	7						
PD5	No	10	2	3	.1834349	1.413231	.59552	.82806	-	[2]
	Yes	19	1	7						
PD6	No	8	1	2	.0854461	0.306644	>.9999	>.9999	-	[2]
	Yes	21	2	8						
PD7	No	10	1	5	.1371726	0.790285	.75690	.82806	-	[2]
	Yes	19	2	5						
PD8	No	15	0	0	.4990412	10.459770	**.00309**	**.04326**	PL vs EN .0107	[2]
	Yes	14	3	10						
PD9	No	20	3	5	.2590719	2.818966	.26585	.53170	-	[2]
	Yes	9	0	5						
PD10	No	13	1	3	.1333604	0.746970	.76891	.82806	-	[2]
	Yes	16	2	7						
PD11	No	13	3	4	.2937168	3.623323	.16338	.38122	-	[2]
	Yes	16	0	6						
PD12	No	11	3	6	.3452317	5.005768	.08401	.29404	-	[2t]
	Yes	18	0	4						
PD13	No	15	2	3	.2113919	1.876834	.44897	.78570	-	[2]
	Yes	14	1	7						
PD14	No	3	0	2	.1616550	1.097558	.72080	.82806	-	[2]
	Yes	26	3	8						

Legend: PL – Polish, FR – French, EN – English, [#] Benjamini-Hochberg correction for multiple comparisons, [&] uncorrected, [2] Fisher's χ^2, φ_c – Cramér's phi, OR – odds ratio, CI – confidence interval, [t] tendency (p = 0.05 – 0.1); *p*-values < 0.05 are indicated in bold.

Table S15. Comparison of communication domain (CD) between analyzed children in different languages in group with ASD.

		PL	FR	EN	φ_c	χ^2	*p*-value	*p*-value corr[#]	*p*-value sub-differences[&]	
CD1	No	18	0	2	.4422088	8.213041	**.01374**	.07695	PL vs EN .0309	2
	Yes	11	3	8						
CD2	No	1	0	0	.0753778	9.025338	.93144	.93144	-	2
	Yes	21	0	3						
CD3	No	2	1	3	.3161773	4.198659	.12254	.24508	-	2
	Yes	27	2	7						
CD4	No	2	0	2	.2082683	1.821779	.46135	.55362	-	2
	Yes	27	3	8						
CD5	No	21	1	5	.2661553	2.975223	.2259	.33885	-	2
	Yes	8	2	5						
CD6	No	7	3	3	.4176654	7.326662	**.02565**	.07695	PL vs FR .0409	2
	Yes	22	0	7						

Legend: PL – Polish, FR – French, EN – English, [#] Benjamini-Hochberg correction for multiple comparisons, [&] uncorrected, [2] Fisher's χ^2, φ_c – Cramér's phi, OR – odds ratio, CI – confidence interval, [t] tendency ($p = 0.05 – 0.1$); *p*-values < 0.05 are indicated in bold.

Table S16. Comparison of social skill domain (SS) between analyzed children in different languages in children with DDX3X syndrome.

		PL	FR	EN	φ_c	χ^2	*p*-value	*p*-value corr[#]	*p*-value sub-differences[&]	
SS1	No	2	9	12	.1429350	1.430129	.48916	.65221	-	[2]
	Yes	4	12	31						
SS2	No	1	3	3	.1288166	1.161560	.55946	.67135	-	[2]
	Yes	5	18	40						
SS3	No	1	1	0	.2843982	5.661765	.05896	.27916	-	[2t]
	Yes	5	20	43						
SS4	No	0	3	2	.1882031	2.479428	.28947	.49623	-	[2]
	Yes	6	18	41						
SS5	No	1	5	20	.2476512	4.293178	.11688	.35064		[2]
	Yes	5	16	23						
SS6	No	3	11	22	.0140118	0.013743	.99315	.99315	-	[2]
	Yes	3	10	21						
SS8	No	0	0	0	N/A	N/A	N/A		N/A	-
	Yes	4	10	20						
SS9	No	6	19	26	.3559873	8.870889	**.01185**	.14220	FR vs EN **.0136**	[2]
	Yes	0	2	17						
SS10	No	2	15	29	.2122525	3.153578	.20664	.41328	-	[2]
	Yes	4	6	14						
SS11	No	0	3	6	.1176938	0.969628	.61581	.67179	-	[2]
	Yes	6	18	37						
SS12	No	1	10	18	.1626160	1.851077	.39632	.59448	-	[2]
	Yes	5	11	25						
SS13	No	5	10	15	.2758005	5.324612	.06979	.27916	-	[2t]
	Yes	1	11	28						
SS14	No	1	4	17	.2207621	3.411513	.18164	.41328	-	[2]
	Yes	5	17	26						

Legend: PL – Polish, FR – French, EN – English, [#] Benjamini-Hochberg correction for multiple comparisons, [&] uncorrected , [2] Fisher's χ^2, N/A – not available, φ_c – Cramér's phi, OR – odds ratio, CI – confidence interval, [t] tendency ($p = 0.05$ – 0.1); *p*-values < 0.05 are indicated in bold.

Table S17. Comparison of play domain (PD) between analyzed children in different languages in children with DDX3X syndrome.

		PL	FR	EN	φ_c	χ^2	*p*-value	*p*-value corr[#]	*p*-value sub-differences[&]	
PD1	No	1	19	15	.5392959	20.358804	.00004	.00056	PL vs FR .0019<br>FR vs EN <.0001	2
	Yes	5	2	28						
PD2	No	1	2	3	.0975102	0.665577	.71692	.82012	-	2
	Yes	5	19	40						
PD3	No	0	3	1	.2433035	4.143763	.12595	.27886	-	2
	Yes	6	18	42						
PD4	No	2	1	3	.2731527	5.222868	.07343	.23330	-	2t
	Yes	4	20	40						
PD5	No	1	1	10	.2203403	3.398490	.18282	.31994	-	2
	Yes	5	20	33						
PD6	No	1	3	1	.2372572	3.940370	.13943	.27886	-	2
	Yes	5	18	42						
PD7	No	1	5	7	.0882231	0.544833	.76154	.82012	-	2
	Yes	5	16	36						
PD8	No	1	14	21	.2664628	4.970170	.08332	.23330	-	2t
	Yes	5	7	22						
PD9	No	1	6	14	.0972730	0.662342	.71808	.82012	-	2
	Yes	5	15	29						
PD10	No	1	5	7	.0882231	0.544833	.76154	.82012	-	2
	Yes	5	16	36						
PD11	No	2	9	16	.0616375	0.265943	.87549	.87549	-	2
	Yes	4	12	27						
PD12	No	1	8	22	.2072048	3.005367	.22253	.34616	-	2
	Yes	5	13	21						
PD13	No	0	11	7	.4122857	11.898562	.00261	.01827	FR vs EN .0026	2
	Yes	6	10	36						
PD14	No	0	5	20	.3119774	6.813092	.03316	.15475	FR vs EN .0085	2
	Yes	6	16	23						

Legend: PL – Polish, FR – French, EN – English, [#] Benjamini-Hochberg correction for multiple comparisons, [&] uncorrected , [2] Fisher's χ^2, φ_c – Cramér's phi, OR – odds ratio, CI – confidence interval, [t] tendency (p = 0.05 – 0.1); *p*-values < 0.05 are indicated in bold.

Table S18. Comparison of communication domain (CD) between analyzed children in different languages in children with DDX3X syndrome.

		PL	FR	EN	φ_c	χ^2	*p*-value	*p*-value corr[#]	*p*-value sub-differences[&]	
CD1	No	4	10	20	.1113009	0.867153	.64819	.99799	-	[2]
	Yes	2	11	23						
CD2	No	0	1	9	.1441130	2.521357	.51063	.87238	-	[2]
	Yes	4	9	20						
CD3	No	0	1	5	.1446170	1.463986	.48095	.99799	-	[2]
	Yes	6	20	38						
CD4	No	0	4	8	.1393714	1.359708	.50669	.99799	-	[2]
	Yes	6	17	35						
CD5	No	2	10	17	.0892220	0.557240	.75683	.99799	-	[2]
	Yes	4	11	26						
CD6	No	1	6	11	.0704337	0.347264	.84061	>.9999	-	[2]
	Yes	5	15	32						

Legend: PL – Polish, FR – French, EN – English, [#] Benjamini-Hochberg correction for multiple comparisons, [&] uncorrected , [2] Fisher's χ^2, φ_c – Cramér's phi, OR – odds ratio, CI – confidence interval, [t] tendency (p = 0.05 – 0.1); *p*-values < 0.05 are indicated in bold.

Table S19. Comparison of age in social skill domain (SS) between analyzed children.

		N	Me	Q1	Q3	*U*	Z-score	*p*-value	*p*-value corr#	
SS1	No	33	10	7	13	1089.0	-1.36582	.17200	.87458	1
	Yes	79	8	5	12					
SS2	No	20	9	6	12	894.0	-0.193724	.84639	.97640	1
	Yes	92	9	6	13					
SS3	No	7	10	9	11	281.0	-1.03373	.30830	.89992	2
	Yes	105	9	5	13					
SS4	No	17	11	6	13	747.0	-0.486538	.62949	.94914	2
	Yes	95	9	6	12					
SS5	No	51	9	5	11	1229.5	-1.90175	.05721	.50682	1t
	Yes	61	9	7	16					
SS6	No	44	10	6	13	1295.5	1.191518	.23345	.89992	1
	Yes	68	8	5	11					
SS8	No	10	11	9	13	401.5	-1.10198	.27271	.89992	2
	Yes	102	9	5	12					
SS9	No	77	9	6	13	1328.0	-0.119268	.90506	.97640	1
	Yes	35	9	6	12					
SS10	No	41	9	5	11	1279.5	-1.06000	.28914	.89992	1
	Yes	71	10	7	13					
SS11	No	14	6	5	11	547.0	1.218496	.22567	.89992	2
	Yes	98	9	6	13					
SS12	No	51	10	6	13	1323.5	-1.35255	.17620	.87458	1
	Yes	61	9	5	11					
SS13	No	46	8	5	11	1321.5	1.159195	.24638	.89992	1
	Yes	66	9	6	13					
SS14	No	32	7	5	11	951.0	2.115762	.03437	.36531	1
	Yes	80	10	6	13					

Lagend: N – number of cases, Me – Median, Q1-Q3 – lower and upper quartile, # Benjamini-Hochberg correction for multiple comparisons, [1] Mann-Whitney *U* test, [2] Mann-Whitney *U* two-tailed test, [t] tendency (p = 0.05 – 0.1); *p*-values < 0.05 are indicated in bold.

Table S20. Comparison of age in play domain (PD) between analyzed children.

		N	Me	Q1	Q3	*U*	Z-score	*p*-value	*p*-value corr#	
PD1	No	59	10	7	15	1047.5	-3.00411	**.00266**	**.03666**	1
	Yes	53	7	5	10					
PD2	No	16	9	6	10	746.5	0.174612	.85946	.99949	2
	Yes	96	9	6	13					
PD3	No	13	9	5	10	569.5	0.667651	.50579	.99651	2
	Yes	99	9	6	13					
PD4	No	15	10	4	11	697.5	0.252024	.79971	.99949	2
	Yes	97	9	6	13					
PD5	No	85	10	6	13	1040.0	0.727853	.46670	.99651	1
	Yes	27	8	5	12					
PD6	No	16	10	7	13	682.5	-0.706764	.48070	.99651	2
	Yes	96	9	6	13					
PD7	No	29	9	6	13	1083.5	-0.793745	.42734	.99621	1
	Yes	83	9	6	12					
PD8	No	51	10	7	13	1114.0	-2.57656	**.00998**	.12224	1
	Yes	61	8	5	10					
PD9	No	49	9	6	11	1495.5	-0.278597	.78055	.99949	1
	Yes	63	8	6	13					
PD10	No	30	9	5	13	1149.5	-0.525622	.59915	.99651	1
	Yes	82	9	6	12					
PD11	No	47	9	6	13	1353.5	-1.02293	.30634	.98211	1
	Yes	65	8	5	11					
PD12	No	51	9	5	13	1551.5	0.020449	.98369	.99949	1
	Yes	61	9	6	12					
PD13	No	38	9	5	11	1371.5	-0.208941	.83450	.99949	1
	Yes	74	9	6	13					
PD14	No	30	7	4	13	981.5	1.629429	.10322	.72947	1
	Yes	82	10	6	12					

Legend: N – number of cases, Me – Median, Q1-Q3 – lower and upper quartile, # Benjamini-Hochberg correction for multiple comparisons, [1] Mann-Whitney *U* test, [2] Mann-Whitney *U* two-tailed test; *p*-values < 0.05 are indicated in bold.

Table S21. Comparison of age in communication domain (CD) between analyzed children.

		N	Me	Q1	Q3	*U*	Z-score	*p*-value	*p*-value corr#	
CD1	No	58	8	4	11	1259.0	-1.78472	.07431	.32027	1t
	Yes	54	9	7	13					
CD2	No	53	16	11	21	1139.5	-2.21637	.02667	.14971	1
	Yes	57	7	4	11					
CD3	No	12	7	5	9	442.0	1.481635	.13985	.38320	2
	Yes	100	9	6	13					
CD4	No	16	6	3	11	577.5	1.579826	.11379	.38320	2
	Yes	96	9	6	13					
CD5	No	56	9	5	13	1511.5	-0.325875	.74452	.74452	1
	Yes	56	9	6	13					
CD6	No	81	9	5	11	1095.5	-1.03726	.29961	.50946	1
	Yes	31	9	6	13					

Legend: N – number of cases, Me – Median, Q1-Q3 – lower and upper quartile, # Benjamini-Hochberg correction for multiple comparisons, [1] Mann-Whitney *U* test, [2] Mann-Whitney *U* two-tailed test, [t] tendency ($p = 0.05 – 0.1$); *p*-values < 0.05 are indicated in bold.

Table S22. Comparison of age in social skills domain (SS) between analyzed children with ASD.

		N	Me	Q1	Q3	*U*	Z-score	*p*-value	*p*-value corr#	
SS1	No	10	10	9	13	128.5	-0.915468	.35796	.98146	[2]
	Yes	32	9	6	12					
SS2	No	13	10	6	11	188.0	0.00000	>.9999	>.9999	[2]
	Yes	29	10	7	12					
SS3	No	5	10	9	11	86.50	-0.213616	.82131	.99898	[2]
	Yes	37	10	6	13					
SS4	No	12	11	8	13	126.0	-1.48956	.13784	.83088	[2]
	Yes	30	9	6	11					
SS5	No	7	10	6	11	102.5	-0.658124	.50797	.99302	[1]
	Yes	35	9	7	14					
SS6	No	10	10	7	13	119.0	1.196014	.23582	.93208	[2]
	Yes	32	8	6	10					
SS8	No	0	-	-	-	-	-	-	-	-
	Yes	70	10	6	12					
SS9	No	26	9	5	11	150.5	1.476334	.13765	.83088	[2]
	Yes	16	11	9	13					
SS10	No	17	10	6	12	199.0	0.333124	.74180	.99885	[2]
	Yes	25	9	7	11					
SS11	No	5	7	6	13	91.50	-0.019420	.96999	.99910	[2]
	Yes	37	10	7	11					
SS12	No	22	10	7	13	197.0	-0.566647	.57095	.99376	[1]
	Yes	20	9	6	11					
SS13	No	16	10	7	12	202.5	-0.129503	.88803	.99898	[2]
	Yes	26	10	6	12					
SS14	No	10	8	6	13	132.0	0.812109	.42276	.98767	[2]
	Yes	32	10	7	12					

Legend: N – number of cases, Me – Median, Q1-Q3 – lower and upper quartile, # Benjamini-Hochberg correction for multiple comparisons, [1] Mann-Whitney *U* test, [2] Mann-Whitney *U* two-tailed test

Table S23. Comparison of age in play domain (PD) between analyzed children with ASD.

		N	Me	Q1	Q3	*U*	Z-score	*p*-value	*p*-value corr#	
PD1	No	24	10	7	12	181.0	-0.876867	.38456	.98733	2
	Yes	18	9	5	12					
PD2	No	10	9	6	10	130.0	0.871171	.38957	.98733	2
	Yes	32	10	7	13					
PD3	No	9	10	6	10	140.0	0.245227	.80961	.99946	2
	Yes	33	10	7	12					
PD4	No	9	10	4	10	111.0	1.134175	.26175	.96959	2
	Yes	33	10	7	13					
PD5	No	27	10	9	13	149.0	1.391249	.16602	.92127	2
	Yes	15	9	6	11					
PD6	No	11	10	7	11	160.5	-0.271771	.77804	.99946	2
	Yes	31	9	6	13					
PD7	No	16	10	7	11	202.0	0.142453	.88803	.99946	2
	Yes	26	10	6	13					
PD8	No	15	11	7	13	156.5	-1.19437	.23021	.96667	2
	Yes	27	9	6	11					
PD9	No	28	10	7	11	193.5	0.053363	.94749	.99946	2
	Yes	14	10	6	13					
PD10	No	17	10	7	13	194.5	-0.448435	.64813	.99827	2
	Yes	25	10	6	11					
PD11	No	20	10	8	13	182.0	-0.944412	.34496	.98546	1
	Yes	22	10	6	11					
PD12	No	20	10	7	12	198.5	-0.528871	.59690	.99827	1
	Yes	22	9	6	12					
PD13	No	20	10	7	11	219.0	0.012592	.98995	.99946	1
	Yes	22	10	6	13					
PD14	No	5	13	7	14	62.50	-1.14576	.25255	.96959	2
	Yes	37	10	6	11					

Legend: N – number of cases, Me – Median, Q1-Q3 – lower and upper quartile, # Benjamini-Hochberg correction for multiple comparisons, [1] Mann-Whitney *U* test, [2] Mann-Whitney *U* two-tailed test

Table S24. Comparison of age in communication domain (CD) between analyzed children with ASD.

		N	Me	Q1	Q3	*U*	Z-score	*p*-value	*p*-value corr#	
CD1	No	22	11	7	13	200.5	0.478502	.63229	.98097	1
	Yes	20	9	6	11					
CD2	No	1	-	-	-	-	-	-	-	-
	Yes	24	11	7	13					
CD3	No	6	8	7	10	94.0	0.485247	.63581	.98097	2
	Yes	36	10	6	13					
CD4	No	4	9	4	17	70.5	0.214242	.82026	.98097	2
	Yes	38	10	7	12					
CD5	No	15	10	7	13	168.0	0.892499	.37645	.90573	1
	Yes	27	9	6	11					
CD6	No	29	9	6	11	170.0	-0.489732	.62858	.98097	2
	Yes	13	10	7	13					

Legend: N – number of cases, Me – Median, Q1-Q3 – lower and upper quartile, # Benjamini-Hochberg correction for multiple comparisons, [1] Mann-Whitney *U* test, [2] Mann-Whitney *U* two-tailed test

Table S25. Comparison of age in social skills domain (SS) between analyzed children with DDS3X.

		N	Me	Q1	Q3	*U*	Z-score	*p*-value	*p*-value corr#	
SS1	No	23	9	6	15	461.5	-0.981563	.32632	.78799	1
	Yes	47	8	5	12					
SS2	No	7	9	5	15	214.5	-0.107673	.90853	.90853	2
	Yes	63	8	5	13					
SS3	No	2	15	9	21	36.0	-1.11046	.29897	.78799	2
	Yes	68	8	5	13					
SS4	No	5	5	5	5	109.0	1.208636	.23471	.78799	2
	Yes	65	9	6	13					
SS5	No	44	6	4	10	374.5	-2.39450	**.01664**	.18242	1
	Yes	26	10	7	16					
SS6	No	34	9	6	16	562.0	0.581669	.56079	.80709	1
	Yes	36	8	5	11					
SS8	No	0	-	-	-	-	-	-	-	-
	Yes	70	8	5	13					
SS9	No	19	9	6	15	373.5	1.459358	.14368	.78799	2
	Yes	51	7	4	10					
SS10	No	24	8	5	11	443.0	-1.34248	.17944	.78799	1
	Yes	46	10	7	16					
SS11	No	9	5	5	9	191.0	1.456308	.14745	.78799	2
	Yes	61	9	6	13					
SS12	No	29	9	6	16	481.0	-1.34725	.17790	.78799	1
	Yes	41	8	4	11					
SS13	No	40	7	4	11	483.0	-1.38260	.16679	.78799	1
	Yes	30	9	6	16					
SS14	No	22	7	5	10	381.0	1.853391	.06383	.51592	1t
	Yes	48	9	6	16					

Legend: N – number of cases, Me – Median, Q1-Q3 – lower and upper quartile, # Benjamini-Hochberg correction for multiple comparisons, [1] Mann-Whitney *U* test, [2] Mann-Whitney *U* two-tailed test; *p*-values < 0.05 are indicated in bold.

Table S26. Comparison of age in play domain (PD) between analyzed children with DDS3X.

		N	Me	Q1	Q3	*U*	Z-score	*p*-value	*p*-value corr[#]	
PD1	No	35	11	6	16	373.5	-2.80144	**.00509**	.06892	1
	Yes	35	7	4	10					
PD2	No	6	10	2	16	188.5	-0.062939	.94295	.99980	2
	Yes	64	8	5	13					
PD3	No	4	4	3	13	81.0	1.277765	.21002	.92522	2.
	Yes	66	9	5	13					
PD4	No	6	10	9	11	156.0	-0.744773	.46601	.99647	2.
	Yes	64	8	5	13					
PD5	No	58	10	5	12	318.0	-0.459705	.64975	.99957	2
	Yes	12	8	5	13					
PD6	No	5	9	5	15	147.0	-0.342067	.73998	.99957	2
	Yes	65	8	5	12					
PD7	No	13	9	5	21	311.0	-0.891055	.37723	.99122	2
	Yes	57	8	5	11					
PD8	No	36	10	6	16	408.5	-2.38543	**.01706**	.20044	1
	Yes	34	7	4	10					
PD9	No	21	9	5	13	513.5	-0.006408	.99489	.99980	1
	Yes	49	8	6	12					
PD10	No	13	9	5	19	353.5	-0.249193	.79973	.99957	2
	Yes	57	8	6	12					
PD11	No	43	9	5	16	539.0	0.494686	.62082	.99957	1
	Yes	27	8	5	11					
PD12	No	31	9	5	13	569.0	0.413825	.67900	.99957	1
	Yes	39	8	6	13					
PD13	No	18	8	5	21	462.0	0.073907	.94162	.99980	2
	Yes	52	9	6	12					
PD14	No	25	7	4	9	390.5	2.102080	**.03555**	.35230	1
	Yes	45	10	6	15					

Legend: N – number of cases, Me – Median, Q1-Q3 – lower and upper quartile, [#] Benjamini-Hochberg correction for multiple comparisons, [1] Mann-Whitney *U* test, [2] Mann-Whitney *U* two-tailed test; *p*-values < 0.05 are indicated in bold.

Table S27. Comparison of age in communication domain (Cd) between analyzed children with DDS3X.

		N	Me	Q1	Q3	*U*	Z-score	*p*-value	*p*-value corr[#]	
CD1	No	36	6	4	10	409.0	-2.37956	**.01733**	.08372	[1]
	Yes	34	10	7	14					
CD2	No	1	-	-	-	-	-	-	-	[1]
	Yes	33	6	4	10					
CD3	No	6	6	2	8	106.5	1.783260	.07232	.25940	[2t]
	Yes	64	9	5	14					
CD4	No	12	5	3	10	248.5	1.542737	.12203	.32323	[2]
	Yes	58	9	6	13					
CD5	No	41	7	5	11	500.5	-1.11476	.26495	.45971	[1]
	Yes	29	9	6	13					
CD6	No	18	9	4	10	386.5	1.088450	.27612	.45971	[2]
	Yes	52	8	6	13					

Legend: N – number of cases, Me – Median, Q1-Q3 – lower and upper quartile, [#] Benjamini-Hochberg correction for multiple comparisons, [1] Mann-Whitney *U* test, [2] Mann-Whitney *U* two-tailed test, [t] tendency (p = 0.05 – 0.1) ; p-values < 0.05 are indicated in bold.
