# Supplementary material for: First Diagnostic Questionnaire for Assessing Patients’ Social Functioning: Comprehensive DDX3X Syndrome Patient Profile
Source: J Clin Med. 2024 Dec 22;13(24):7842. doi: 10.3390/jcm13247842 (PMC11676840; doi:10.3390/jcm13247842)
Supplement: Supplementary file 1 [file jcm-13-07842-s001.zip › 07.08.2024 Supplementary 2 - Social behaviour questionnaire by Stefaniak.pdf]

Social behavior questionnaire by Stefaniak

BASIC INFORMATION ABOUT YOUR CHILD:

1. What is your child’s gender?
a. Female
b. Male
c. Other

2. What is your child’s age? Please write the month and year of your child’s birth.

………………………………………

3. Where are you from?
a. North America
b. South America
c. Australia
d. Asia
e. Europe
f. Africa

Country of your origin:………………..

MEDICAL DOMAIN (MD_1-MD_10)

MD_1 Does your child have a diagnosis of Autism Spectrum Disorder (ASD)?
a. Yes
b. No

MD_2 Does your child have a diagnosis of DDX3X Syndrome?
a. Yes
b. No

MD_3 Is your child experiencing early life feeding difficulties or ongoing chronic constipation?
a. Yes
b. No

MD_4 Does your child have cardiac and/or respiratory problems (e.g. sleep apnea, congenital heart disease, etc.)?
a. Yes
b. No

MD_5 Is your child experiencing sensory integration (SI) difficulties?
a. Yes
b. No

MD_6 Does your child have a high pain threshold (for example, your child only starts to feel pain when it is at a high level, such as a very loud sound, a very high temperature or a very intense squeeze)?
a. Yes
b. No

MD_7 Does your child have motor stereotypies, hyperactivity, or physical aggression?
a. Yes
b. No

MD_8 Does your child engage in self-injurious behaviours (SIBs, e.g. head-hitting, self-pinching, banging his or her head against the wall, etc.)?
a. Yes
b. No

MD_9 Does your child have facial dysmorphism (for example, wide nasal bridge and narrow nasal wings at the same time)?
a. Yes
b. No

MD_10 Does your child have microcephaly?
a. Yes
b. No

MD_11 Is your child using AAC? Do części medycznej
a. Yes
b. No

MD_12 What type of AAC?
a. tablet/speech synthesizer
b. sign language
c. pictograms
d. other

SOCIAL SKILLS (SS_1-SS_14)

SS_1 Was your child “happy” and non-problematic in the first year of life?
a. Yes
b. No

SS_2 Would you describe your child as being social with family, friends, and people in general?
a. Yes
b. No

SS_3 Does your child smile or laugh often and/or have a good sense of humour?
a. Yes
b. No

SS_4 Would you describe your child as a “good-natured child” who is caring with a friendly personality?
a. Yes
b. No

SS_5 Would you describe your child as having a strong desire to make friends/ befriending?
a. Yes
b. No

SS_6 Can your child develop and sustain friendships?
a. Yes
b. No

SS_7 Does your child experience heightened self-consciousness in social situations?
a. Yes
b. No

SS_8 Does your child recognize relatives and friends?
a. Yes
b. No

SS_9 Do you notice your child recognizing people he/she met only once before? Zmień punktację w ostatecznej wersji na 1p-Tak, 0p-Nie
a. Yes
b. No

SS_10 Would you recognize the emotion of “shy” as social behaviour and a reaction of your child to the social environment of friends, family, and strangers (e.g., Your child is sharing a moment or a good laugh with someone but is feeling shame because someone is looking at them so they are shy and they are smiling at the same time; Your child is feeling shame because they recognize a friend they haven’t seen for a while and they recognized them so they are feeling shame, covering their face and smiling at the same time etc.)?
a. Yes
b. No

SS_11 Does your child enjoy receiving public praise? (for example, does he or she like to be applauded, complimented, and/or praised for something they have done, and can you notice your child's enjoyment of that?)
a. Yes
b. No

SS_12 Does your child present with a willingness to persevere in the face of difficulties or challenges?
a. Yes
b. No

SS_13 Does your child have anxiety-related behaviours, including social withdrawal, noticeable worries about changes in routines, or unusual fears of common sounds and objects (for example: onions, animals in stripes)?
a. Yes
b. No

SS_14 Is the quality of emotions adequate to the context of the situation- is your child overreacting or underreacting in social situations?
a. Yes
b. No

PLAY DOMAIN (PD_1-PD_14)

PD_1 Does your child have the ability to spontaneously explore the environment by themself (for example, trying new routes during walks, being excited to visit new places, trying to listen to new music, etc.)?
a. Yes
b. No

PD_2 Is your child fascinated with water?
a. Yes
b. No

PD_3 Does your child love to swim?
a. Yes
b. No

PD_4 Does your child love to listen to music?
a. Yes
b. No

PD_5 Does your child love to dance?
a. Yes
b. No

PD_6 Does your child enjoy it when family members and/or friends stay together at the table, are in one room, play with each other, dance, or do fun activities together?
a. Yes
b. No

PD_7 Is your child interested in what other children are doing (for example, do they like to play with other children or watch what other kids are playing with and/or copy what they are doing)?
a. Yes
b. No

PD_8 Would you describe your child as a cognitively gifted child (for example, even if they are nonverbal, have low functional communication, and/or have more difficulties understanding the natural contingencies in their environment)?
a. Yes
b. No

PD_9 Is your child getting easily into play/interaction with other children and wanting to be involved even if they lack play skills and don't know the rules of the game, still wish to participate and be among them?
a. Yes
b. No

PD_10 Is your child interested in other children (for example, smiling at them, observing them, approaching them)?
a. Yes
b. No

PD_11 Is your child often trying to attract your attention with their " performances" and trying to get verbal praise?
a. Yes
b. No

PD_12 Is your child like psychical activity/group activity?
a. Yes
b. No

PD_13 Is your child initiating play or talking with you spontaneously?
a. Yes
b. No

PD_14 Does your child have an extraordinary memory for details?
a. Yes
b. No

COMMUNICATION (CD_1-CD_8)

CD_1 Is your child verbal (using words and/or sentences)?
a. Yes
b. No

CD_2 If your child is nonverbal, do you see any attempts from them to use functional communication through gestures and sounds and/or to look deeply into your eyes or the eyes of other people?
a. Yes
b. No
c. Non-applicable – my child communicates verbally

CD_3 Does your child experience difficulties with expressing needs, including difficulties in describing emotions or problems in explaining what they need in a moment however, you can notice a strong will in your child to be understood yet lacking abilities to be fully understood?
a. Yes
b. No

CD_4 Does your child have speech understanding? For instance, when you ask your child to do the task "put the book on a table" or "bring me the pillow," will they be able to do this without you pointing a finger at the object?
a. Yes
b. No

CD_5 If something new happens, is your child looking at your face to see how you are feeling about that? For example, when they play with a relative they haven't seen for a long time and want to check your feelings about that, or when they hear a strange or funny sound, will they check your face before reacting?
a. Yes
b. No

CD_6 Is your child pointing to the thing they want, but it's out of their range?
a. Yes
b. No

Please name your child's strengths.
…………………………………………………………………………………………..

Additional comments:
………………………………………………………………………………………………...
…………………………………………………………………………………………………
………….……………………………………………………………………………………...
…………………………………………………………………………………………………
…………………………………………………………………………………………………

Thank you very much for your time.
