# Supplementary material for: First Diagnostic Questionnaire for Assessing Patients’ Social Functioning: Comprehensive DDX3X Syndrome Patient Profile
Source: J Clin Med. 2024 Dec 22;13(24):7842. doi: 10.3390/jcm13247842 (PMC11676840; doi:10.3390/jcm13247842)
Supplement: Supplementary file 1 [file jcm-13-07842-s001.zip › 07.08.2024 Supplementary 3 - Scoring of questionnaire questions.pdf]

SCORING OF QUESTIONNAIRE QUESTIONS:

MD (MEDICAL DOMAIN)

There is no scoring in this domain of the questionnaire.

Questions MD_9 and MD_10 were separately scored if they have had an influence to other results in other domains.

Questions MD_11 and MD_12 receive no scoring.

SS (SOCIAL SKILLS)

Question	Positive answer (“Yes”)	Negative answer (“No”)
SS_1	1	0
SS_2	1	0
SS_3	1	0
SS_4	1	0
SS_5	1	0
SS_6	1	0
SS_7	-	-
SS_8	1	0
SS_9	0	1
SS_10	1	0
SS_11	1	0
SS_12	1	0
SS_13	0	1
SS_14	0	1

SS_7 was excluded from the questionnaire.

PD (PLAY DOMAIN)

Question	Positive answer (“Yes”)	Negative answer (“No”)
PD_1	1	0
PD_2	1	0
PD_3	1	0
PD_4	1	0
PD_5	1	0
PD_6	1	0
PD_7	1	0
PD_8	0	1
PD_9	1	0
PD_10	1	0
PD_11	1	0
PD_12	1	0
PD_13	1	0
PD_14	0	1

CD (COMMUNICATION DOMAIN)

Question	Positive answer (“Yes”)	Negative answer (“No”)
CD_1	0	1
CD_2	1	0
CD_3	1	0
CD_4	0	1
CD_5	1	0
CD_6	1	0

CD_2 has had an additional answer, “Non-applicable”, and in this case there is no scoring
